# Strategic approaches for developing a culture of safety management in schools: Indications from literature studies

**DOI:** 10.4102/jamba.v11i2.694

**Published:** 2019-07-04

**Authors:** Sithulisiwe Bhebhe, Tawanda Runhare, Ratau J. Monobe

**Affiliations:** 1 Faculty of Education, University of Swaziland, Kwaluseni, Swaziland; 2 School of Education, University of Venda, Thohoyandou, South Africa

**Keywords:** Natural Disasters, Disaster Preparedness, Disaster Management, Post-Disaster Therapy, Child Security, Disaster Prevention, Civic Education

## Abstract

Natural disasters can take away children’s lives and their right to quality education. This article identifies and discusses strategies that schools can employ to prepare for and minimise the effects of natural disasters. Using theoretical propositions and literature on disaster management, the article discusses strategies for the prevention of and preparedness to respond to and recovery from natural disasters within a school setting. Evidence from research indicates that there are basic principles and practices of disaster management that school management and learners may not be aware of. Based on the identified theoretical principles and practices for disaster management, the article concludes that both state and non-state parties have disaster management responsibilities and therefore should formulate and disseminate the basic principles and practices of disaster prevention, preparedness and post-disaster therapy to schools because of the vulnerability of children to disaster. In addition, it also recommends that disaster management should be included in the school curricula through subjects like geography, science, social studies or civic education and life orientation or skills training.

## Introduction

Natural disasters are a worldwide phenomenon that adversely affects many different things in a country and ultimately prevents the smooth running of essential services, particularly those that affect children. According to Wilson et al. (2007:1), ‘[*d*]isasters are natural or man-made emergency events which have negative economic and social consequences for the affected population’. Cardona (2003) states that these emergencies are termed disasters when they occur and the losses exceed the capacity of the population to support or resist it. When storms, extreme temperatures, fire, earthquakes, cyclones, typhoons, droughts, floods and desertification occur, millions of children are prevented from attending school (Lazarus, Jimerson & Brock 2003). As a result of natural catastrophe, schools should be alert to receive information when a disaster is coming and communicate it to the school-based educational stakeholders and communities so that they can get ready and be safe from any occurrence of natural disasters, should they occur. Apart from the home, schools are another primary habitat where children spend most of their time; therefore, schools should promote a culture of safety. According to NDMC SA. (2015), all disaster management centres in cities and districts are required to play an active role in engaging schools to ensure a practical approach to disaster awareness programmes.

In most cases, learners are susceptible to natural disasters; hence, it is important that teachers and school managers consider planning for the prevention and management of these disasters that might occur in the area where their schools are located. This is in view of the fact that there are some disasters that are caused by human error such as fire, electrical faults and drowning. In this regard, Pasipamire (2011) advises that the level of preparedness and the methods used in the class to teach disaster management principles and practices has a greater positive impact on the lives of the learners and teachers.

Natural disasters not only physically distract but also traumatise the learners. The effects of disasters such as earthquakes, veld fires and violent floods are petrifying, especially for schoolchildren, because of their vulnerability at times of disaster. The schools’ learning programmes become undesirably affected by natural disasters and at times result in temporary termination of teaching and learning. In this regard, Lazarus et al. (2003) also advise that schools should play an important role in providing a stable and familiar environment when disasters occur. Interruptions in the school routines may result in some learners dropping out of school altogether. In such a scenario, those children that could be academically gifted and keen to learn could become disillusioned and lose the impetus to learn.

## A documentary survey of effects of natural disasters on schools

While a number of countries have taken into consideration the prevention, preparedness and management of natural disasters, there is still a need to provide safety measures upon the occurrence of disasters. In New Zealand, there is a disaster preparedness policy that came into effect after the impacts of the 2011 Christchurch earthquake, which resulted in 185 deaths and significant damage to property and human life (GNS Science 2011). Schoolchildren are amongst those that may be affected by death through natural disasters.

In the United States of America (USA), the Council on School Health (2008) notes that studies focusing on recent natural disasters have concurred that there are several important deficiencies in school preparedness for emergencies. This prompted the US policymakers to come up with policy guidelines that redressed the identified deficits in the schools’ disaster preparedness. Developing a culture of safety management will ensure that schools are prepared for any kind of disasters that are likely to occur in their area.

In the context of Africa, in recent years, recurrent floods have befallen Mozambique since 2000 and these floods have exposed the sub-Saharan region of its disaster unpreparedness. Persistent rainfall accompanied by tropical cyclone Eline caused excessive flows in rivers, such as the Limpopo, which has catchments in other countries like Zambia and Zimbabwe. These floods affected a total of about 4.5 million people and caused over 700 deaths; losses were estimated to be US$500 million and the Gross Domestic Profit (GDP) growth rate is estimated to have declined from 10% to 2%. Making reference to the effect of these Mozambican disaster situations on schools, Luis (2014) observes that:

Disasters have caused a disruption of education in Mozambique for quite some time. In 2012, Cyclone Funso and Tropical Storm Dando damaged 1000 classrooms along the eastern coastline and in 2013 heavy flooding affected 250 classrooms in the Limpopo Basin. (p. 3)

In Africa, consistent floods have initiated convocation in education in Mozambique and neighbouring countries. Floods have been continuously damaging the schools and the surrounding environment and communities, leaving schoolchildren without schools and some even without homes (Luis 2014:3).

In Ethiopia, the most serious floods reoccurred in May 1968, August 1994 and May 2005, causing significant damage estimated to be US$93 512m, and affecting the lives of about 3.5 million people (OFDA/CRED Centre for Research on the Epidemiology of Disasters Emergency Events Database 2002). From 2004 to 2006, flooding afflicted several areas of eastern and southern Ethiopia, Somalia and Kenya, killing and displacing hundreds of people. The Shabelle and Juba rivers in the region have flooded their banks, affecting towns and villages in an area stretching across hundreds of kilometres during floods. Floods in the Horn of Africa normally follow the June–September rainy season in most years. According to one United Nations (UN) report, the 2006 floods, which followed droughts in 2005, affected 1.8 million people and were the worst in the region in the last 50 years (ICSU 2007). Disasters have affected the lives of people on the African continent, including damage to schooling infrastructure, meaning that some children’s education gets delayed while the schools are being fixed and this takes up expense from the country’s economy.

The International Centre of Technology (2014) states that in Kenya the frequency of disasters has increased rapidly over the recent years. Reports on Africa regarding natural disasters have registered an increase over the past decades (OFDA-CRED 2002). Therefore, this calls for an urgent need for African schools in Africa to come up with clear and urgent policies and strategies that are responsive to this human threat. The occurrences of natural disasters in Africa call for preparedness, particularly in schools.

Despite the massive damage that natural and man-made disasters have on Africa’s development, minute efforts are given to prevent them. The Council on School Health (2008) advises that schools must have disaster plans that are uniquely designed in terms of provision. There is also a need for schools to have resources and expertise to implement disaster management.

Natural disasters also have economic and social bearings on learners, their families and communities. In South Africa, after a severe storm in the Northern Cape province, a report by the Northern Cape Office of the Premier (2011:23) indicated that ‘key areas that have been severely affected in the province are Agriculture, Tourism, Education, Human Settlements in addition to direct and indirect citizens’. In other words, because of essential services getting affected in the occurrence of such disasters, the normal life of both adults and children gets disturbed.

## The compulsion for disaster management in schools

Schools provide a second home to the children and should therefore be hospitable, habitable and provide security to children. A culture based on safety should be promoted and practiced in schools so as to provide an assurance of disaster security (NDMC S.A. 2015). In this regard, Warfield (2015) observes that disaster management in general reduces or avoids human and material losses from disasters, assures prompt and appropriate assistance to victims of a disaster and leads to rapid and effective recovery from a disaster. It is thus mandatory that schools fully prepare themselves for the possibilities of natural disasters for the safeguard of their staff, learners, teachers and key resources and infrastructure.

Generally, the greatest socialising institution for learners after the family is the school. This calls for schools to demonstrate the need to be prepared for all hazardous possibilities in the event of natural disasters, acts of terrorism and the threat of pandemic diseases ((Murray & Choo 2005). However, experiences in Africa in a café suggest that the greatest challenge in Africa is that most societies react to disasters instead of being proactive on preventive measures. For example, some schools’ geographical locations and infrastructure setup do not have disaster prevention mechanisms, which is an indication of inadequate consideration of disasters in the planning process. In this regard, Luis (2014) observes that:

Given the large economic and social costs involved as consequence of the occurrence of cyclones, floods, windstorms and other adverse climate phenomena, recently aggravated by the effect of global climate change, we have to take more appropriate measures so that our schools are safer. (p. 3)

An important step in planning is to conduct situational needs identification and analysis of every school based on the geographical setup, community trends and previous accident and disaster data. To address environmental disasters such as toxic spills, hurricanes, tornadoes, floods and earthquakes, schools should have infrastructure suitable for natural disasters that are likely to occur (Murray 2005). In this case, the landscape or terrain where schools are built should not be low-lying areas like valleys. Drainage systems should always be well catered for the architectural plan and also well maintained. Making reference to Africa, the UN-Habitat Global Activities Report (2015) indicates that most schools in Mozambique are sited in areas that can be exposed to one or more natural disasters and 200 – 1000 classrooms have been found to be affected by cyclones and floods every time floods occurred. The high impact is often because of issues such as poor structural design, use of subpar construction materials and *ad hoc* building practices (ICSU 2007). The above observations buttress the need to plan school infrastructures with disaster possibilities in mind. Infrastructural planners should avoid schools that are sandwiched by rivers that make schools not easily accessible during rainy season, as this causes danger to learners who cross these rivers going to school every day. Simple issues like earthing rods to curb lightning strikes are often overlooked. For disaster management to develop into a culture in schools, teachers and learners should be initiated on the subject of disaster consciousness and prevention. Teachers and learners should also be equipped with school-based safety precautions, policies and practices as well as intervention plans for when disaster strikes.

## Disaster preparedness within a school setting

Education is vital in preparing and managing disasters. Most developed countries, such as Japan and USA, use education to prepare children and their families for natural disasters. Disaster preparedness is meant to achieve a satisfactory level of readiness to respond to any emergency situation. In preparing for a disaster, there is a need to ensure that strategic reserves of food, equipment, water, medicines and other essentials are in place. Disaster preparedness goes a long way in reducing the impact of natural predicaments on education (Greubel, Ackerman & Winthrop 2012).

Preparing for a disaster entails that governmental and non-state parties develop plan procedures to save lives, minimise disaster damages and enhance disaster response operations. Preparedness plans for schools should include emergency exercises or training, warning systems, emergency communications systems, evacuation plans, resource inventories, emergency personnel contact lists, mutual aid agreements and public information systems (Warfield 2015). This entails that all new members of staff and first-entering students should be inducted on the institutional disaster prevention and management policies and procedures.

The level of disaster preparedness varies according to the socio-economic status of a continent or nation. For example, in Japan, despite the fact that the great 8.9 magnitude earthquake destroyed lives of teachers and children, classes commenced in a week after the disaster because of the post-disaster mechanisms that were in place. Disaster-proof and multi-hazard resilient buildings had been erected as an infrastructure coping strategy for disasters (Greubel et al. 2012). According to Wilson et al. (2007), African countries are lagging behind developed countries (such as the USA) when it comes to disaster preparedness. As a result, Africa continues to experience more effects of disasters, which expose schoolchildren to be vulnerable to disaster strike.

In preparing for a disaster, there should be broad participatory involvement in order to save lives as well as infrastructure. Each school should have a crisis management team that works with the community members involved in crisis planning and management to assess the medical equipment, mental health and other resources available in the school. Schools should also identify children with special healthcare needs and put in place practical emergency care plans. There should be evacuation chairs that can glide down stairwells for children in wheelchairs and visually and hearing impaired children should be assigned to members of cluster crisis team. Staff and schoolchildren need to be educated on the multiple evacuation routes so that they can use those in the occurrence of a disaster, and such escape ways should be plotted on a diagram displayed on every school building (ICSU 2007; Council on School Health 2008).

## Disaster preparedness mechanisms for schools

School disaster planning is a facet of larger community planning and, therefore, requires coordinated planning and allocation of community resources. Promptly responding to a disaster provides immediate assistance to preserve life, improve health and support those affected by the disaster. According to Warfield (2015), immediate response to a disaster entails meeting the basic needs of people until more permanent and sustainable solutions can be found. Plans should be developed in partnership with other community groups, including law enforcement, fire safety, public health, Emergency Medical Services (EMS), and paediatric and mental health professionals. During the response for a disaster, it is important to identify children who could have challenges to cope with and address any developing mental health concerns. Paediatricians, other healthcare professionals such as first-aid responders, public health officials, the media, school nurses, school staff and parents all need to be united in their efforts to support schools in the prevention of, preparedness for, response to and recovery from a disaster (Council on School Health 2008). A teamwork approach is therefore strongly recommended for both planning and implementation of disaster interventions.

There are departments in some countries that are entrusted with the responsibility to provide services like counselling for the family members who have lost their loved ones as a direct consequence of disasters and community members affected by the disaster, supply of food parcels, home-cooked meals that can be accessed through the soup kitchens, tents for temporary shelter, blankets, mattresses and clothing. Therefore, there is a need for funding to be set aside at school level so that it could be made available during disasters to avoid crisis management or being reactionary to disaster situations. In the case of South Africa, the South African Social Security Agency has made funding available from their Social Relief of Distress Fund whenever disasters occur, and this has helped to overcome catastrophes (Social Assistance Act 13 2004).

In countries where floods cause inaccessibility via roads, community members may be air-lifted from the disaster damaged areas. There is also a need that after a disaster, emergency teams are put on high alert at schools for cases of diarrhoea, cholera, malaria or any other medical crisis that can occur as a result of floods. For example, in South Africa, after a flood occurred in the Northern Cape province in 2011, a mobile clinic was set up at Blaauwskop in the Upington region to assist in case there was an outbreak of diseases as a result of the floods (Northern Cape Office of the Premier 2011). In Zimbabwe, when cholera struck its bordering town with South Africa, a non-governmental humanitarian group called Doctors without Borders set up clean water supply points and assisted the infected to fight the epidemic in 2009 after the government health structures had been overwhelmed.

According to Lazarus et al. (2003), after a disaster there should be collaboration between the school crisis response team and the different community, state and national organisations and agencies to respond to the diverse needs of the children in schools. Maintaining close contact with teachers and parents, the school crisis response team should determine which students need supportive crisis intervention and counselling services after a disaster (Lazarus et al. 2003). It is vital for schools to be aware of children with chronic diseases so that in response to a disaster, medication for chronic patients could be made available and dispatched to such children.

Regular monitoring and testing of water after a disaster is important to ensure that children are safe from water-borne diseases. Schoolchildren and the community have to be educated on water purification, good sanitation, hygienic food preparation, treatment formula, diarrhoea and handwashing and be warned against drinking water from rivers or any flooded pools. Medical conditions of people evacuated from a disaster may include asthmatic cases, diarrhoea, diabetes, trauma, allergic reactions, hypertension, stroke, fever, epilepsy and respiratory problems, as experienced in one South African region (Northern Cape Office of the Premier 2011). The Council on School Health (2008) proposed that school district officials and mental health professionals must be readily available in all disaster recovery centres to disseminate information and provide guidance and help to set up school-based disaster management structures.

## Disaster recovery strategies for schools

From the school perspective, disaster recovery means restoring the infrastructure in the school and ensuring that teaching and learning resume in the shortest possible time. Learners recover rapidly after a disaster when concrete explanations of what happened and how it could or will affect them are provided and dealt with (Council on School Health 2008). Collaboration between the school crisis response team and an assortment of community, state and national organisations and agencies is necessary to respond to the many needs of children, families and communities following a natural disaster. While the aftermath of a natural disaster takes time to heal, especially in learners, good and well-structured disaster preparedness and prompt response facilitate a speedy recovery from a disaster (Lazarus et al. 2003). Such recovery response mechanisms include counsellor, scholar, food, clothing and mechanical provisions by multifaceted professionals.

After a disaster, returning to the classroom does not always ascertain that children are ready to engage with their learning tasks. Warfield (2015) advises that recovering from a disaster requires those affected by the disaster to restore their lives and the damaged infrastructure first. There is a need for support from parents and school personnel to help children return to normal activities, routines and provide learners with an opportunity to talk about the frightening event, such as a healing experience (Lazarus et al. 2003). Community and school structures have a responsibility to support with the necessary mental health resources and determine which therapies are appropriate for the school to attain some form of healing and be able to operate normally (Council on School Health 2008).

### Post-disaster therapy

There are a number of ways which could be implemented to assist schools that have been psychologically and emotionally affected by natural disasters. These include supporting teachers and other school staff, healing activities, disaster-related discussions, problem-solving skills, children’s friendship and peer support, children’s resiliency, crisis response team and mental health support systems (Lazarus et al. 2003). This implies that a multisectorial team of professionals needs to be identified and involved in undertaking the following and other post-disaster therapy and rehabilitation accomplishments.

#### Staff support system:

- There should be provision to teaching and non-teaching school staff members with information on the symptoms of children’s stress reactions and guidance on how to handle class discussions and answer children’s questions. Teachers should pay attention to their own needs and should not feel compelled to do anything they are not comfortable with. Time for staff members to share their feelings and reactions should be on a voluntary basis. In addition, staff members who suffered property damage or personal injuries may need leave from work to attend to such needs after a natural disaster. This means that management should be flexible in monitoring school routines and programmes after a disaster.

#### Post-disaster healing process:

- There is also a need to engage in post-disaster activities that facilitate healing. The healing activities include exposure to discussion of disaster-related events, promotion of positive coping and problem-solving skills and strengthening of children’s friendship and peer support.

#### Open discussions:

- Children should be encouraged to talk about disaster-related events so as to steam off. They need an opportunity to discuss their experiences in a safe and supportive environment. In addition, children may be assigned activities that enable them to discuss their experiences. These may include a range of methods, both verbal and non-verbal, and incorporate varying projects, for example, drawing, stories, and audio- and video recording.

#### Problem-solving:

- Promotion of problem-solving skills helps children cope with the aftermath of a disaster. Children should be engaged in activities that assist them to solve disaster-related stressors such as family walks and group games. They should be encouraged to develop realistic and positive methods for coping with stress that increase their ability to manage their anxiety and to identify those mechanisms that fit in each situation.
- Children with strong emotional support from others are better able to cope with this adversity. Relationships with peers can provide suggestions for how to cope with difficulties and can help decrease isolation. In many disaster situations, friendships may be disrupted because of family relocations. In some cases, parents may be less available to provide support to their children because of their own distress and their feelings of being overwhelmed. It is important for children to develop supportive relationships with their teachers and classmates. Activities may include asking children to work cooperatively in small groups in order to enhance peer support.

#### Resistance coping strategies:

- There is a need to emphasise on children’s resilience so as to come to terms with the negative impacts of natural disasters. Focus should be on their competencies in terms of their daily life and in other difficult times. They should be helped to identify what they have done in the past that helped them cope when they were frightened or upset.
- All crisis response team members need an opportunity to process the crisis response. Providing crisis intervention is emotionally draining because a lot has to be considered, like tone of voice, pitch and facial expressions. This is likely to include teachers and other school staff if they have been serving as crisis caregivers for students.

Although more than enough caregivers are often willing to provide support during the immediate aftermath of a natural disaster, long-term services may be lacking. School psychologists and other school mental health professionals can help provide and coordinate mental health services, but it is important to connect with community resources in order to provide long-term assistance. Ideally, these relationships should be established in advance for disaster preparedness.

### Ethical Considerations

Data used in this study was collected from literature and all ideas taken from literature we acknowledged. No direct harm to people can therefore be influenced by the paper.

## Conclusion

Evidence from the literature indicates that to some extent there could be prevention of, preparedness for, responses to and recovery from disasters within a school setting. The study also reveals that there are basic principles and practices of disaster management that school management and learners may not be aware of and that there is a need to educate learners, teachers and school administrators as well as communities on possible disasters that may affect their community.

Based on observations from the literature on how some disasters have been responded to by different nations, this article concludes that the socio-economic development of a country or a community determines the level of success in disaster preparedness, interventions and post-disaster therapy. The article concludes from available literature on natural disasters, especially in Africa, that schoolchildren are more vulnerable to psychological and emotional trauma during a natural disaster. Therefore, the article argues that effective disaster preparedness and management require collaboration between all stakeholders in order to contribute to the welfare and safety of schoolchildren. This calls for the establishment of school-based disaster management structures with adequate knowledge base on principles and practices of disaster management within a school setting.

This article recommends that there is a need to include disaster management as a substantive subject in the school curriculum which should empower learners with knowledge regarding safety in schools. Furthermore, subjects like geography, physical science, life skills and social studies should incorporate key topics on disasters prevention, interventions and post-recovery. Schools, particularly in Africa, should establish disaster management teams, be supplied with effective equipment and knowledge to react to disasters and there should be thorough environmental analysis on where schools should be located. School billboards should be erected in every building. Finally, refresher courses and mock disaster reaction rehearsals should be periodically conducted in schools.
